# Complete Genome Sequence of the Collinolactone Producer *Streptomyces* sp. Strain Gö40/10

**DOI:** 10.1128/mra.00220-22

**Published:** 2022-07-27

**Authors:** Eva B. Sterndorff, Tue S. Jørgensen, Jaime Felipe Guerrero Garzón, Julian Schmid, Stephanie Grond, Tilmann Weber

**Affiliations:** a The Novo Nordisk Foundation Center for Biosustainability, Technical University of Denmark, Kongens Lyngby, Denmark; b Institut für Organische Chemie, Eberhard Karls Universität Tübingen, Tübingen, Germany; Indiana University, Bloomington

## Abstract

The actinomycete *Streptomyces* sp. strain Gö40/10 has the potential to produce a range of secondary metabolites, one of which is collinolactone, a compound with neuroprotective properties and potential for pharmaceutical applications. The genome was sequenced with Oxford Nanopore Technologies MinION and Illumina MiSeq systems and consists of a single 9,635,564-nucleotide linear chromosome.

## ANNOUNCEMENT

*Streptomyces* sp. strain Gö40/10 was isolated from soil collected in Bolivia in 1991 and grown in medium NL 8711, which is used routinely for agar slopes (1). This isolate has the ability to produce various complex secondary/specialized metabolites, including collinolactone (2–4), which exhibits neuroprotective properties through inhibition of intracellular oxidative stress on glutamate-sensitive cells and hence has potential as a drug against Alzheimer’s disease (4). Furthermore, *Streptomyces* sp. strain Gö40/10 was described to produce cineromycins and ansamycins (3) and potentially other complex polyketides or peptides. To get access to the biosynthetic gene clusters (BGCs) encoding these biosynthesis pathways, the whole-genome sequence of *Streptomyces* sp. strain Gö40/10 was determined.

The strain was grown in yeast extract-malt extract (YEME) medium with 3.4% sucrose at 30°C with shaking at 250 rpm (5). Purification of genomic DNA (gDNA) was performed by using the Qiagen Genomic-tip 100 kit (Venlo, Netherlands) with prolonged lysis, i.e., 2 h of incubation with B1 and enzyme at 37°C followed by 2 h with B2 at 50°C.

A library was constructed using the rapid barcoding kit (SQK-RBK004; Oxford Nanopore Technologies, Inc.) and run with a FLO-MIN106D (R9.4.1) flow cell on a MinION device. Base calling was done using Guppy base caller (v5.0.11+2b6dbff) in high-accuracy mode followed by demultiplexing by the Guppy barcoder. Adaptors were trimmed from Nanopore reads using Porechop (v0.2.4) (6), and reads smaller than 1,000 nucleotides (nt) were removed using Filtlong (v0.2.0) (7). After trimming, reads with a total of 930,546,501 nt and an *N*_50_ value of 16,263 nt were used for *de novo* assembly with Flye (v2.9-b1768) with the setting --nano-hq and a total of five polishing iterations (8).

Illumina MiSeq data were generated from a KAPA HyperPlus library (Roche, Switzerland), with size selection of approximately 450 to 600 nt, resulting in 2.3 million clusters of read length 2 × 151 bp. Illumina reads were trimmed using Trim Galore with Cutadapt (v2.10), applying the settings --length 100 and --quality 20 (9).

Illumina data were aligned to the Nanopore assembly using bowtie2-align (v2.3.4.1), with an overall alignment rate of 97.08% (10). Finally, the Nanopore assembly was polished with the Illumina data using the Unicycler (v0.4.8) polishing module, resulting in 4,429 changes in base positions (11).

The topology of the genome was determined from the Flye assembly graph and consisted of a single, linear chromosome with a total assembly length of 9,635,564 nt with terminal inverted repeats of 59,464 nt, a GC content of 72.33%, and mean Nanopore coverage of 95×. Gö40/10 was classified by GTDB-tk (v1.7.0) (12) as a member of Streptomyces cinerochromogenes, and the closest autoMLST (v231601f) (13) match was Streptomyces reticuli (GenBank accession number LN997842.1) at 92.8% average nucleotide identity (ANI). The benchmarking universal single-copy orthologs (BUSCO) score (14) was 100% complete BUSCO genes (v4.0.5, actinobacteria_class_odb10), with 3 genes existing in duplicate. PGAP (v2021-11-29.build5742) was used for annotation and yielded 8,226 coding sequences (CDSs), of which 7,950 were functionally annotated, with 7 rRNA operons and 72 tRNAs.

Analysis with antiSMASH (v6.0.0alpha1-0bc1c66) (15) with the parameters --cb-general --cb-subclusters, and --cb-knownclusters revealed 39 regions containing secondary metabolite BGCs, including 3 non-ribosomal peptide synthetase (NRPS), 7 polyketide, 4 terpene, and 6 hybrid BGCs.

### Data availability.

The data are available under NCBI BioProject accession number PRJNA721311. Raw reads were deposited in the SRA under BioSample accession numbers SAMN18970196 (Illumina) and SAMN18970196 (Nanopore). The genome sequence can be found under GenBank accession number CP084203.1.
